# Regulatory of miRNAs in tri-lineage differentiation of C3H10T1/2

**DOI:** 10.1186/s13287-022-03205-3

**Published:** 2022-11-22

**Authors:** Wei Huang, Xiaoyue Wu, Shuaixi Xiang, Mingxin Qiao, Hanfei Li, Yujie Zhu, Zhou Zhu, Zhihe Zhao

**Affiliations:** State Key Laboratory of Oral Diseases & National Clinical Research Center for Oral Diseases, West China Hospital of Stomatology, Sichuan University, Chengdu, 610041 People’s Republic of China

**Keywords:** Adipogenic differentiation, Chondrogenic differentiation, C3H10T1/2, MicroRNA, Osteogenic differentiation

## Abstract

MicroRNAs (miRNAs) are non-coding single-stranded RNA molecules encoded by endogenous genes, which play a vital role in cell generation, metabolism, apoptosis and stem cell differentiation. C3H10T1/2, a mesenchymal cell extracted from mouse embryos, is capable of osteogenic differentiation, adipogenic differentiation and chondrogenic differentiation. Extensive studies have shown that not only miRNAs can directly trigger targeted genes to regulate the tri-lineage differentiation of C3H10T1/2, but it also can indirectly regulate the differentiation by triggering different signaling pathways or various downstream molecules. This paper aims to clarify the regulatory roles of different miRNAs on C3H10T1/2 differentiation, and discussing their balance effect among osteogenic differentiation, adipogenic differentiation and chondrogenic differentiation of C3H10T1/2. We also review the biogenesis of miRNAs, Wnt signaling pathways, MAPK signaling pathways and BMP signaling pathways and provide some specific examples of how these signaling pathways act on C3H10T1/2 tri-lineage differentiation. On this basis, we hope that a deeper understanding of the differentiation and regulation mechanism of miRNAs in C3H10T1/2 can provide a promising therapeutic method for the clinical treatment of bone defects, osteoporosis, osteoarthritis and other diseases.

## Introduction

C3H10T1/2, isolated from mouse embryos, is the primary cell component of loose connective tissue. It is differentiated from mesenchymal cells in the embryonic stage [1]. C3H10T1/2 exhibits strong functional activities, weak basophilic cytoplasm, obvious protein synthesis and secretion activities and is quite vital for the repair of different degrees of cell degeneration and necrosis as well as tissue defects [2]. Recent studies have shown that C3H10T1/2 harbors the potential and ability to differentiate into a variety of cellular types, like chondrocyte osteoblasts and adipose cells [3]. The differentiation of C3H10T1/2 is strictly controlled by its microenvironmental factors, and changes in signaling and molecules may lead to abnormal differentiation [4]. miR-335-5p, miR-21 and other signal channels have been evidenced to have the ability to induce C3H10T1/2 osteogenic differentiation, which will induce the increase in osteoblasts in vivo, imbalance between osteogenesis and osteoclasts, secretion of bone matrix, bone matrix mineralization and formation of new bones [5, 6]. miRNAs, like miR-223 and miR-30e, are closely related to cell adipogenesis [7, 8]. C3H10T1/2 usually differentiates into adipogenic precursor cells first, and under the influence of specific cellular stimulation, such as C/EBPs and PPAR-γ, it eventually differentiates into adipocytes, with the appearance of significant markers such as fat droplets in adipocytes. miR-135a-1-3p, miR-134-5p, and so on have been confirmed to be correlated with the chondrogenic differentiation of C3H10T1/2. They can promote the differentiation of C3H10T1/2 into chondroblast and further differentiate into mature chondrocytes. The cell products and the mesenchymal around the cell mass will differentiate into perichondrium [9, 10]. At present, the differentiation of tri-lineage of C3H10T1/2 is a research hotspot, whereas substantial potentially associated signal channels have not been determined yet.

The differentiation of tri-lineage of C3H10T1/2 depended on the spatiotemporal differences in gene expression of cells, which was not only determined by the internal developmental program of cells, but also influenced and regulated by the extracellular environment, and sometimes such external control conditions or environment played a decisive role in the formation of specific cells [11]. miRNAs are 19–25 ncRNA molecules which inhibit or degrade target genes via the interaction with the 3' untranslated region (3'-UTR) of mRNA transcripts [12]. They are vital for RNA silencing and posttranscription modulation of coding genes. miRNAs are key mediators of substantial biological process, like the forming of tissues [13]. In addition to the direct silencing of mRNAs by miRNAs, miRNAs can also induce tri-lineage differentiation of C3H10T1/2 by acting on different signaling pathways. Wnt signaling pathway is a group of signal transduction pathways of multiple downstream channels stimulated by the binding of ligand protein Wnt and membrane protein receptor, which is extensively involved in cell differentiation, tumorgenesis, brain formation and organogenesis [14]. Mitogen-activated protein kinase (MAPK) is a group of serine-threonine protein kinases that can be activated by various extracellular stimuli such as cytokines, neurotransmitters, hormones, cell stress and cell adhesion. MAPK chain plays a key role in gene expression regulation and cytoplasmic functional activities by transmitting upstream signals to downstream response molecules through sequential phosphorylation [15]. Bone morphogenetic proteins (BMP) are an important member of TGF-β superfamily, and BMPs signaling is transmitted through standard, Smad-dependent, or non-standard pathways. It can significantly induce ectopic bone formation. These three signaling channels all play an important role in stem cell differentiation [16]. Unexpectedly, the above signaling pathways also showed obvious tri-lineage differentiation induction effect in C3H10T1/2, and different results would occur under the regulation of different miRNAs and activation or inhibition of the signaling pathway at different times, which has great research value [17, 18]. At present, the knowledge about the involvement of miRNA in the regulation of C3H10T1/2 differentiation is accumulating. It has been found that a large number of miRNA expressions can interfere with C3H10T1/2 differentiation, but the underlying mechanism has not been fully determined.

In this review, we first identified the key signaling pathways and molecular factors related to the differentiation of C3H10T1/2 tri-lineage and explored how miRNAs participated in and regulated these signaling pathways and factors. In addition, the significance of tri-lineage differentiation of C3H10T1/2 in the early diagnosis of bone defects, prevention of bone loss and bone marrow fat was briefly discussed.

## Biogenesis and functions of miRNA

**Fig. 1:**
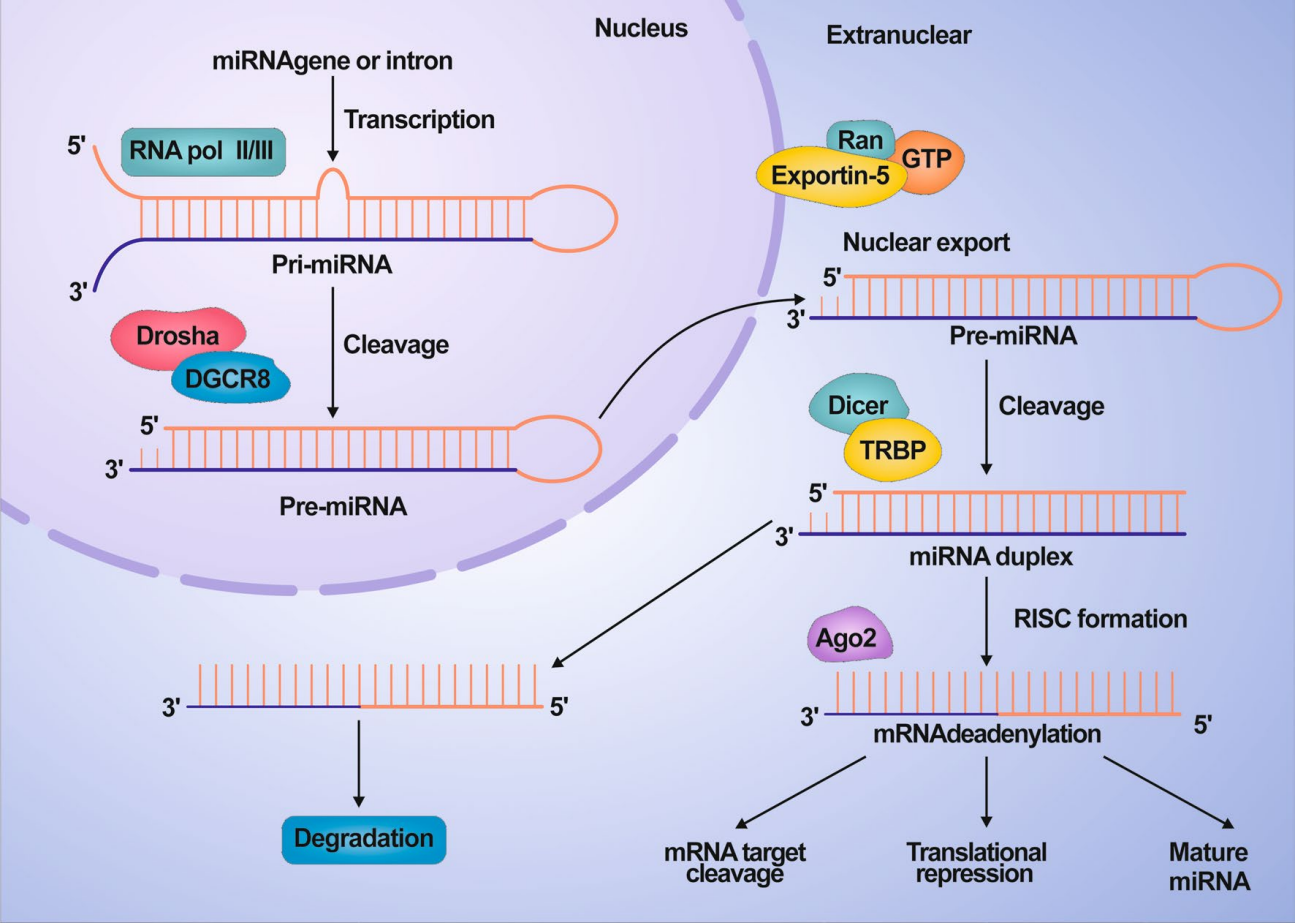
The biogenesis and function of miRNA processing. The maturation of miRNAs includes the production of primary miRNA transcripts (pri-miRNA) by RNA polymerase II or III and cleavage of pri-miRNA in the nucleus by the microprocessor complex Drosha-DGCR8 (Pasha). The resulting precursor hairpin, pre-miRNA, is exported from the nucleus via exportin-5-rang-gtp is exported from the nucleus. In the cytoplasm, the functional strand of the mature miRNA is loaded with Argonaute (Ago2) protein into the RNA-induced silencing complex (RISC), where it directs RISC to silence the target mRNA by mRNA cleavage, translational repression or deadenylation, while the passenger strand is degraded

The biological generation of miRNA is a multi-step process (Fig. 1), starting from the initial transcription of primary miRNA, which has a hairpin/stem-loop architecture [19]. Posterior to the transcription by RNA polymerase II or RNA polymerase III, primary miRNAs are first processed in the nucleus in a sequence-independent but structure-related way by Drosha/DGCR8 microprocessor complex to form precursor miRNAs [20]. Subsequently, the precursor miRNA is in conjunction with exportin-5 at high Ran-GTP levels. Ran/GTP/exportin-5 transports the pre-miRNAs through the nuclear pore complex into the cytoplasm, and the pre-miRNAs are released from the Ran/GTP/exportin-5 complex upon GTP hydrolysis [21, 22]. Then, Dicer collaborates with TRBP to split precursor miRNAs and produce mature miRNAs, which will be incorporated into RISC complexes and afterward bind to the 3'-UTR of the targeted sequence complementarily to cause targeted mRNA decomposition or suppress translational activity [23].

Studies have found that the 5' end of miRNA is more conservative in contrast to the 3' end, and there is a frequently conservative A1 in the targeted mRNA 3′-UTR, adjacent to the downstream sequence of the seed site [24]. A1 is not always capable of binding to the first nucleotide of miRNAs in Watson–Crick base-pair matching, but can be identified by proteins in RISC [25, 26]. Therefore, its mechanism of action can be divided into two types according to the position of nucleotide A on miRNA: the first type predominantly involves the seed site centered at the 5' end of miRNAs, 8MER, 7MER-A1, 7MER-M8 and 7MER sites, which can be referred to as classical miRNA sites [27]. The second type is the 3' supplement site and 3' compensation site, which are at the 3' end of miRNAs, also referred to as non-classical sites [28].

Based on the sequence of the 5' end of miRNAs or the 3' end of miRNAs, interactions between miRNAs and their targeted mRNAs can be forecast [29]. In addition to the classic and the non-classic sites on miRNA, miRNA also observed “seedless” inhibition of mRNA, which is difficult to predict its potential mRNAs that will interact with the miRNA. For instance, E2F2 gene can be straightly suppressed by miR-24 through seedless 3'-UTRs [30]. The whole regulatory mechanism of miRNA is complex and diverse, and a variety of signaling molecules or genes can be involved in regulation [31]. E2F2 gene can be straightly suppressed by miR-24 through seedless 3'-UTRs. In addition, as mentioned above, perfect typical seed loci are not enough to forecast function interactions between miRNAs and their targeted mRNAs. It must be considered that there might be other factors affecting the processes of interactions. Argonaute 2 (AGO) protein is a vital component of RISC complexes [32]. Displayed by structural researches, it shows an elastic architecture that can be stabilized via miRNA binding. Another factor affecting the interplay is the accessibility of the mRNA to the binding site. The binding of the targeted spot on the mRNA to RNA-binding protein (RBP) or the targeted spot contained in the stem-loop structure of the mRNA will prevent the targeted spot from miRNA contact [33]. On the other hand, factors facilitating the releasing of RBP from binding to complementary sequences can produce stem-loop structures with targeted spots, thus increasing the accessibility of the targeted spots of mRNAs [34]. Better binding affinity between RISC and targeted spots can facilitate the releasing of other translation inhibitors from targeted sites and elevate the accessibility [35].

## Regulatory of miRNA in tri-lineage differentiation of C3H10T1/2

### Regulatory role of miRNAs in osteogenic differentiation of C3H10T1/2

Osteogenic differentiation is a key step of bone formation; that is, C3H10T1/2 undergoes a complex process of osteoblast progenitor cells, osteoblast cells and finally osteogenesis [36]. C3H10T1/2 differentiates into osteoblasts under the activation of different miRNAs and other special physicochemical factors [37]. Subsequently, osteoblasts differentiate into osteoblast progenitors and enter a phase of rapid proliferation. Cell proliferation is gradually reduced, and extracellular matrix (ECM) mature-associated genes (alkaline phosphatase, type I collagen, matrix calcitin) are further stimulated [38]. At this stage, osteoblastic cells synthesize and excrete organic matrix to form bones, mainly formed from type I collagen and characterized by alkaline phosphatase [39]. It is an early biomarker of osteoblastic cell differentiation and maturation. Mature osteoblastic cells express ECM calcification-associated proteins, mainly osteocalcin and osteosin, and the mineralizing activity of osteoblastic cells is remarkably elevated [40].

Recently, substantial researches have highlighted the regulation of miRNAs on osteogenetic differentiation of C3H10T1/2 (Table 1). Sun et al. [41] discovered that the down-regulation of miR-138 could promote the osteogenetic differentiation of C3H10T1/2, and that the quantity of ALP-positive cells increased significantly, and that the expression levels of osteogenetic associated genes, like ALP, COL-1, BMP-2, OCN and Runx2, were the most remarkable in cells. Brenner et al. [42] constructed a non-viral osteogenetic genetic therapy vector system, namely BMP-2/miR-590-5p, which displayed the largest elevation in osteogenetic differentiation and more severe mineralization in vitro compared with BMP-2 alone. This hybrid vector provides a new gene therapy strategy for the reconstruction and repair of bone defects. By in vivo and in vitro experiments, Jiao et al. [43] discovered that miR-140-5p overexpression could facilitate the expressing of differentiation-associated genes and calcium deposition in osteogenic medium treated C3H10T1/2 cells, and they detected the increased expressing of osteocalcin, bone mineral density and bone mass in fractured mice, which facilitated bone healing. This might be a treatment target for curing fractures and facilitating osseointegration.

**Table 1:** Regulation of miRNAs in osteogenic differentiation of C3H10T1/2

miRNA	Expression	Target	Expression of target	Distribution	Function	Experiment	Animal model	References
miR-335-5p	↑	DKK1	↓	Cytoplasm	miR-335-5p stimulates Wnt signal transmission and facilitates osteogenetic differentiative activity via the down-regulation of DKK1	In vitro	–	[47]
miR-129-5p	↑	DKK3	↓	Cytoplasm	miR-129-5p promotes the osteogenetic differentiative activity of BMSCs via inhibiting the expressing of DKK3, which interacts with β-catenin directly	In vitro and in vivo	Mice/36	[48]
miR-21a	↑	Smad7	↓	Nucleus	Phytol-treatment promotes osteoblast differentiation via highly expressed Runx2 due to down-regulation of Smad7 by miR-21a	In vitro	–	[6]
miR-140-5p	↑	Runx2, ALP, Spp1 and Bglap3	↑	Nucleus	miR-140-5p promotes osteogenic differentiation via enhancing the expression of osteoblast differentiation-related genes	In vitro and in vivo	Mice/32	[43]
miR-582-5p	↓	Runx2	↑	Cytoplasm	miR‐582‐5p negatively regulates osteogenic differentiation by down-regulating the activity of ALP and mRNA/protein levels of ALP and Runx2	In vitro	–	[49]
miR-590-5p	↑	Smad7	↓	Nucleus	miR-590-5p facilitates the differentiative activity of osteoblasts via straightly targeting Smad7 and non-straightly protecting and stabilizing the Runx2 protein from Smurf2-mediated decomposition	In vitro	–	[50]
miR-144-3P	↓	Smad4	↑	Cytoplasm	miR-144-3p modulates the osteogenetic differentiative and proliferative activity in a negative way of via suppressing the Smad4 protein level	In vitro	–	[51]
miR-324-5p	↓	Gpc1	↑	Cytoplasm	miR-324-5p direct targets Gpc1 and regulates Hedgehog signaling, reducing the content of Ihh and BMP-2-triggered alkaline phosphatase	In vitro	–	[10]
miR-194	↑	COUP-TFII	↓	Cytoplasm	Enforced expressing of miR-194 remarkably reinforces the differentiative activity of osteoblasts, whereas it suppresses the differentiative activity of adipocytes via reducing COUP-TFII mRNA and protein contents	In vitro	–	[52]
miR-125b	↓	Cbfβ	↑	Cytoplasm	miR-125b is a negative modulatory factor of osteogenetic differentiative activity via straightly down-regulating Cbfβ and non-straightly acting on Runx2	In vitro	–	[53]
miR-30a	↓	BMP9	↑	Cytoplasm	miR-30a modulates BMP9-triggered osteogenetic differentiative activity in a negative way	In vitro and in vivo	Mice/44	[45]
miR-433	↓	Runx2	↑	Cytoplasm	miR-433 inhibits BMP-2-triggered osteogenetic differentiative activity via reducing the content of Runx2 transcript	In vitro	–	[46]
miR-223	↓	Fgfr2	↑	Cytoplasm	miR-223 increases FGFR2 protein level and plays an inhibitory role in osteoblast differentiation	In vitro	–	[7]
miR-30e	↓	LRP6	↑	Cytoplasm	miR-30e overexpression negatively affects osteoblast differentiation by targeting LRP6 and the canonical Wnt/β-catenin signal transmission	In vitro	–	[8]

Some target genes may be co-regulated by multiple miRNAs. Runx2 directly stimulates the transcription of mesenchymal cell osteocalcin, type I collagen, bone bridging protein and collagenase 3 genes during osteogenic differentiation. Runx2 is involved in ECM signaling, and when it binds to the osteocalcin promoter, ECM signaling rapidly increases the transcriptional activity of osteocalcin genes through integrin β differentiation toward osteoblasts [44]. Further studies revealed that the overexpression of miR-30a triggered reduced expressing of early osteogenic markers and Runx2 [45]. Kim et al. [46] discovered that ERRγ increased the expression of miR-433 in C3H10T1/2. During the differentiation of osteoblasts, the overexpression of ERRγ or miR-433 repressed the expressing of osteogenetic biomarker genes Runx2 and ALP. Based on this, it can be hypothesized that miR-30a and miR-433 synergistically down-regulate Runx2 gene and inhibit cellular osteogenic differentiation.

In summary, miRNAs have been shown to be closely associated with the osteogenic differentiation of C3H10T1/2 and are a powerful tool in scientific research to reveal osteogenic mechanisms, which can guide the clinical treatment of osteoporosis, bone repair, osteogenesis imperfecta and other genetic diseases.

### Regulatory role of miRNAs in adipogenic differentiation of C3H10T1/2

Adipose tissue consists of adipocytes and is essential for the maintenance of energy and metabolic homeostasis. There are 2 kinds of adipose tissues, one is white adipose tissue (WAT), which is mainly found in subcutaneous and visceral tissues [54]. Pathologically confirmed, visceral white fat is associated with obesity, insulin resistance, and subcutaneous white fat improves glucose tolerance [55]. The other is brown adipose tissue (BAT), which is mainly distributed in discrete forms in the spine, clavicle and paradrenal glands, and its function is to produce heat [56]. Both adipose tissues have the same differentiation characteristics and are derived from mesenchymal stem cells [57]. The adipogenic differentiation of C3H10T1/2 occurs in two stages: First, MSC differentiate into adipogenic progenitor cells and then, under the influence of specific cellular stimuli, such as C/ebp and PPAR-γ, they eventually differentiate into adipocytes and produce significant labeled fat droplets in adipocytes [58, 59]. The phenotypes of white adipose and brown adipocytes can be interconverted. More studies are currently being conducted at the genetic level, including the researches on regulators such as peroxisome proliferator-activated factor receptor γ (PPARγ), PRDM16 containing the PR structural domain and PPARγ coactivator 1α (PGD-1α) [60].

Substantial miRNAs have been shown to be closely related to the adipogenic differentiation of C3H10T1/2 (Table2). Man et al. [61] found that miR-503 overexpression inhibited BMPR1a and adipogenic factors during adipocyte differentiation, such as CCAAT/enhancer binding protein A (C/EBPα), PPAR and adipocyte protein 2 (AP2). The adipogenic activity was decreased by the PI3K/Akt pathway. Studies have shown that brown fat formation induces the upward regulation of miR-485, which interferes with the expressing of SRPK1 via targeting the 3'-UTR of SRPK1. Further, the loss of endogenetic SRPK1 promotes the developmental process of C3H10T1/2 cells into brown fat cells [62].

**Table 2:** Regulation of miRNAs in adipogenic differentiation of C3H10T1/2

miRNA	Expression	Target	Expression of target	Distribution	Function	Experiment	Animal model	References
miR-194	↓	COUP-TFII	↑	Cytoplasm	Enforced expression of miR-194 inhibits adipocyte differentiation by decreasing COUP-TFII mRNA and protein levels	In vitro	–	[52]
miR-140	↑	Ostm1	↓	Cytoplasm	miR-140 overexpression promotes BMP-4-induced adipocyte lineage commitment via decreasing the expression of Ostm1	In vitro	–	[63]
miR-223	↑	Fgfr2	↓	Cytoplasm	miR‐223 promotes adipocyte differentiation through down-regulating Fgfr2	In vitro	–	[7]
miR-30e	↑	LRP6	↓	Cytoplasm	miR-30e overexpression positively affects adipocyte formation by targeting LRP6 and the canonical Wnt/β-catenin signaling	In vitro	–	[8]
miR-27	↓	Lox	↑	Cytoplasm	miR-27 represses adipogenic lineage commitment by inhibiting the expression of Lox	In vitro and in vivo	Mice/20	[64]
miR-1	↑	RBM4	↑	Nucleus	Overexpressing miR-1 enhances brown adipocytes differentiation via up-regulating RBM4 that enhances skipping of the MEF2Cγ region which functions as a transcriptional repressor	In vitro	–	[65]
miR-10a-5p	↑	Rora, Dll4 and Nr4a3	↓	Cytoplasm	miR-10a-5p performs as a positive regulator that reduces adipose tissue inflammation and promotes therapeutic adipogenesis by down-regulating anti-adipogenic molecules	In vitro and in vivo	Mice/68	[66]
miR-195a	↓	Zfp423	↑	Cytoplasm	miR-195a is an anti-adipogenic regulator, which acts by negatively targeting Zfp423	In vitro	–	[67]
miR-20a	↑	KDM6B and TGFBR2	↓	Cytoplasm	miR-20a promotes adipocyte progenitor cells to differentiate and this function may depend upon its inhibitory effects on KDM6B and TGF-β signaling	In vitro	–	[68]
miR-503-5p	↓	Zfp217	↑	Cytoplasm	there was a positive relationship between adipocyte differentiation and Zfp217 expression, which can be suppressed by miR-503-5p, miR-135a-5p and miR-19a-3p	In vitro and in vivo	Mice/12	[69]
miR-582-5p	↑	Runx2	↓	Cytoplasm	miR‐582‐5p induces adipogenic differentiation by down-regulating the activity of ALP and mRNA/protein levels of ALP and Runx2	In vitro	–	[49]
HSA-Let-7 g	↓	FST and TNFRSF4	↑		let-7 g down-regulation may enhance intramuscular adipogenesis during fetal muscle development possibly via down-regulating the mRNA levels of FST and TNFRSF4	In vitro and in vivo	Mice/12	[70]
miR-485	↑	SRPK1	↓	Cytoplasm	brown adipogenesis-induced up-regulation of miR-485 interferes with SRPK1 expression	In vitro	–	[62]

### Regulatory role of miRNAs in chondrogenic differentiation of C3H10T1/2

Cartilage destruction and loss are important pathological changes in many orthopedic diseases, including osteoarthritis [71]. Due to the special anatomical and tissue characteristics of cartilage, such as the lack of vascular and lymphatic distribution, cartilage has an abnormally insufficient ability to repair itself [72]. Therefore, the use of artificial methods to repair damaged cartilage is of great clinical importance. The process of MSC cartilage differentiation is that MSC first retract and aggregate into a group. Intermediate cells are transformed by division and differentiation into large, round cells, known as chondrogenic cells producing mesenchyme and fibers (mainly type II collagen) [73]. When the amount of matrix increases to a certain level, the chondrogenic cells are separated into traps and differentiate into mature chondrocytes [74].

There are increasing studies on the chondrogenic direction of C3H10T1/2 cartilage (Table 3). Wa et al. [75] have found in vivo and in vitro that NFATc1 binds to the SOX9 promoter under hypoxic conditions when the methylation of CpG islands in the promoter down-regulates miR-124, inducing Sox9 expression and promoting the chondrogenic differentiation of C3H10T1/2. miR-199b-5p overexpression inhibits C3H10T1/2 cellular growth, whereas it promotes the transforming growth factor-β3 (TGF-β3)-triggered chondrogenic differentiation of C3H10T1/2 cells. The gene and protein expression of chondrocyte-specific biomarkers, like SOX9, Aggrecan and type II collagen (Col2a1), is enhanced. In contrast, the inhibition of miR-199b-5p significantly promotes C3H10T1/2 cell proliferation but decreases chondrogenic differentiation [76].

**Table 3:** Regulation of miRNA in chondrogenic differentiation of C3H10T1/2

miRNA	Expression	Target	Expression of target	Distribution	Function	Experiment	Animal model	References
miR-135a-1-3p	↓	Hoxd10	↑	Cytoplasm	miR-135a-1-3p is a remarkably differentially expressed miRNA that targets Hoxd10, who is involved in chondrogenesis	In vitro	–	[9]
miR-124	↓	NFATc1	↑	Cytoplasm	miR-124 is down-regulated and NFATc1 is up-regulated undergoing chondrogenesis in hypoxic conditions	In vitro	–	[77]
miR-30b	↓	SOX9	↑	Cytoplasm	miR-30b is a pivotal negative modulator of TGF-β3-triggered chondrogenic differentiation, which functions via straightly inhibiting SOX9	In vitro	–	[75]
miRNA-140	↓	CXCL12	↑	Cytoplasm	miR-140 inhibition promoted MSC viability via targeting and decreasing CXCL12 protein contents during TGF-β3-triggered chondrogenic differentiation	In vitro and in vivo	Mice/18	[78]
miR-199b-5p	↑	JAG1	↓	Cytoplasm	miR-199b-5p is the positive modulators to regulate the chondrogenetic differentiative activity of via down-regulating JAG1	In vitro	–	[76]
miR-145	↓	Sox9	↑	Cytoplasm	miR-145 is a pivotal negative modulator of the chondrogenetic differentiative activity of via straightly inhibiting Sox9 at early phases of chondrogenetic differentiative activity	In vitro	–	[79]

Although there is a large gap in the researches on the mechanism of cartilage formation, they still provide important guidance for the clinical treatment of cartilage injury and other diseases and offer a solid foundation for further exploration.

**Fig. 2:**
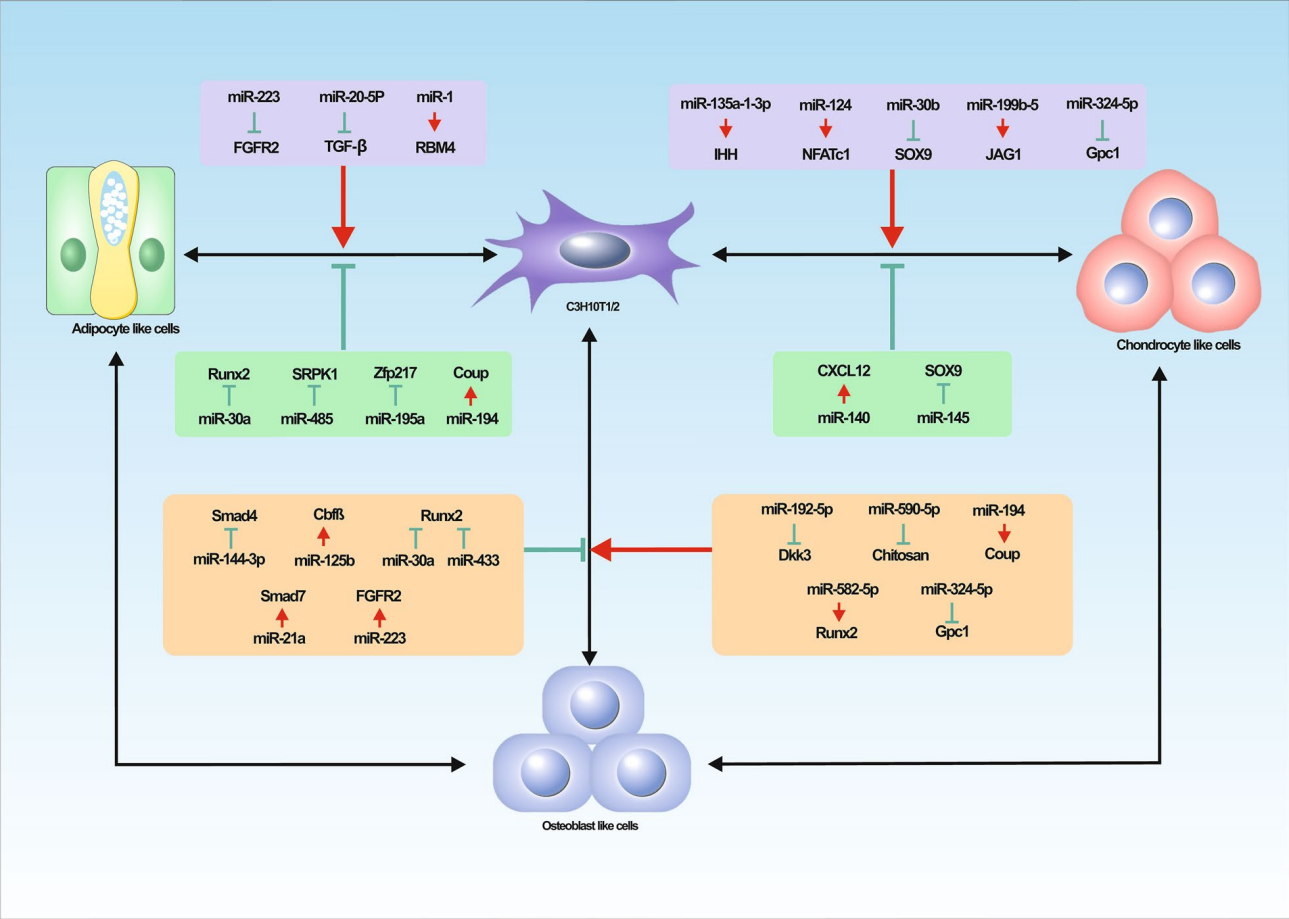
miRNAs expression regulation in tri-lineage differentiation. C3H10T1/2 is capable of osteogenic, adipogenic and chondrogenic differentiation. miRNAs targeted related genes, growth factors, and signal channels by increasing or decreasing their own expression, and further guide the commitment of tri-lineage differentiation. The dynamic balance among osteogenic and adipogenic differentiation, osteogenic and chondrogenic differentiation is regulated by specific miRNAs. “ → ”represents the upstream signal promoting the downstream signal, and “⊥”represents the downstream signal inhibiting the downstream signal

Above all, miRNAs exert a vital modulatory effect on the tri-lineage differentiations of C3H10T1/2 (Fig. 2). We have compiled and initially created a corresponding mechanism network, hoping to provide a reference for the studies on miRNAs regulating cell differentiation and facilitate the further development of this field.

### Regulation of balance among osteogenic, adipogenic and chondrogenic differentiation

A large number of miRNAs have been shown to exert regulatory effects on the triple lineage of C3H10T1/2, and some miRNAs have the ability to regulate both or more than two-way differentiation, of which the balance between osteoblast and adipogenic differentiation and the balance between osteoblast and chondrocyte differentiation are predominant (Table 4).

Both osteoblastic cells and adipose cells are stemmed from bone marrow mesenchymal stem cells, which suggests a balanced differentiation between osteogenesis and adipogenesis in vivo. C/EBPα expression is first up-regulated and then down-regulated during the BMP-2-induced directional differentiation of C3H10T1/2 cells. The overexpression of C/EBPα suppresses the BMP-2-induced osteogenetic differentiation of C3H10T1/2 cells [80]. BMP-2 triggers the differentiation of C3H10T1/2 cells toward osteoblastic cells compared to earlier stages and reduces adipogenic differentiation potential due to the induction of C/EBPα down-regulation in the adipogenic signaling pathway [81]. Moreover, it was found that some miRNAs (miR-223, miR-582-5p, miR-194, miR-30e) can affect both osteogenic and adipogenic differentiation of C3H10T1/2. In most cases, the effects of the same miRNA on osteoblast and adipogenic differentiation are often opposite. When miRNA can significantly induce cell osteogenesis, it also tends to inhibit the adipogenic differentiation of cells and reduce the infiltration of adipocytes, thus providing conditions for the treatment of osteoporosis and steroid-induced femoral head necrosis [52]. On the contrary, if this miRNA has a significant adipogenic differentiation effect, the expression of common osteogenic markers (ALP, Runx2, OCN) in cells is often significantly reduced in the experimental results [7, 8, 49]. Osteoporosis (OP) is mainly caused by decreased osteogenic differentiation and increased adipogenic differentiation [82]. Clinical manifestations include decreased bone mass, increased bone fragility, and the degeneration of bone microarchitecture [83]. A thorough revelation of those signal paths can help to better elucidate the pathogenesis of osteoporosis and provide clinicians with effective treatment regimens for osteoporosis.

Extensive studies have revealed the role of chondrogenic osteoblast transdifferentiation in bone tissue formation, suggesting a balance between osteogenic differentiation and the chondrogenic differentiation of C3H10T1/2. Although the exact cause of the conversion of chondrocytes to osteoblasts is elusive, this conversion requires regulatory factors. For example, the Runx2 gene is a major regulator of osteogenic fate and can transdifferentiate adipocytes, primary adult skeletal muscle cells and adult dentin vascular smooth muscle cells into osteoblast types [84]. Runx2 enhances chondrocyte proliferation by inducing Indian porcupine protein (Ihh), and the high expression of Ihh induces parathyroid hormone-like hormone production that inhibits Runx2 and chondrocyte maturation, leading to a negative feedback regulation of chondrocyte maturation [85]. Imbalance in the regulation of Runx2 may contribute to the development of osteoarthritis. However, in the current cell experiments involving C3H10T1/2, it is relatively clear that miR-199a has the potential to promote osteogenic differentiation and chondrogenic differentiation at the same time. Studies have shown that miR-199a was up-regulated in osteoblasts and chondrocytes differentiation, while miR-96 expression was increased during osteogenesis and adipogenesis, but not during chondrogenesis. miR-124 was exclusively expressed in adipocytes [86].

**Fig. 3:**
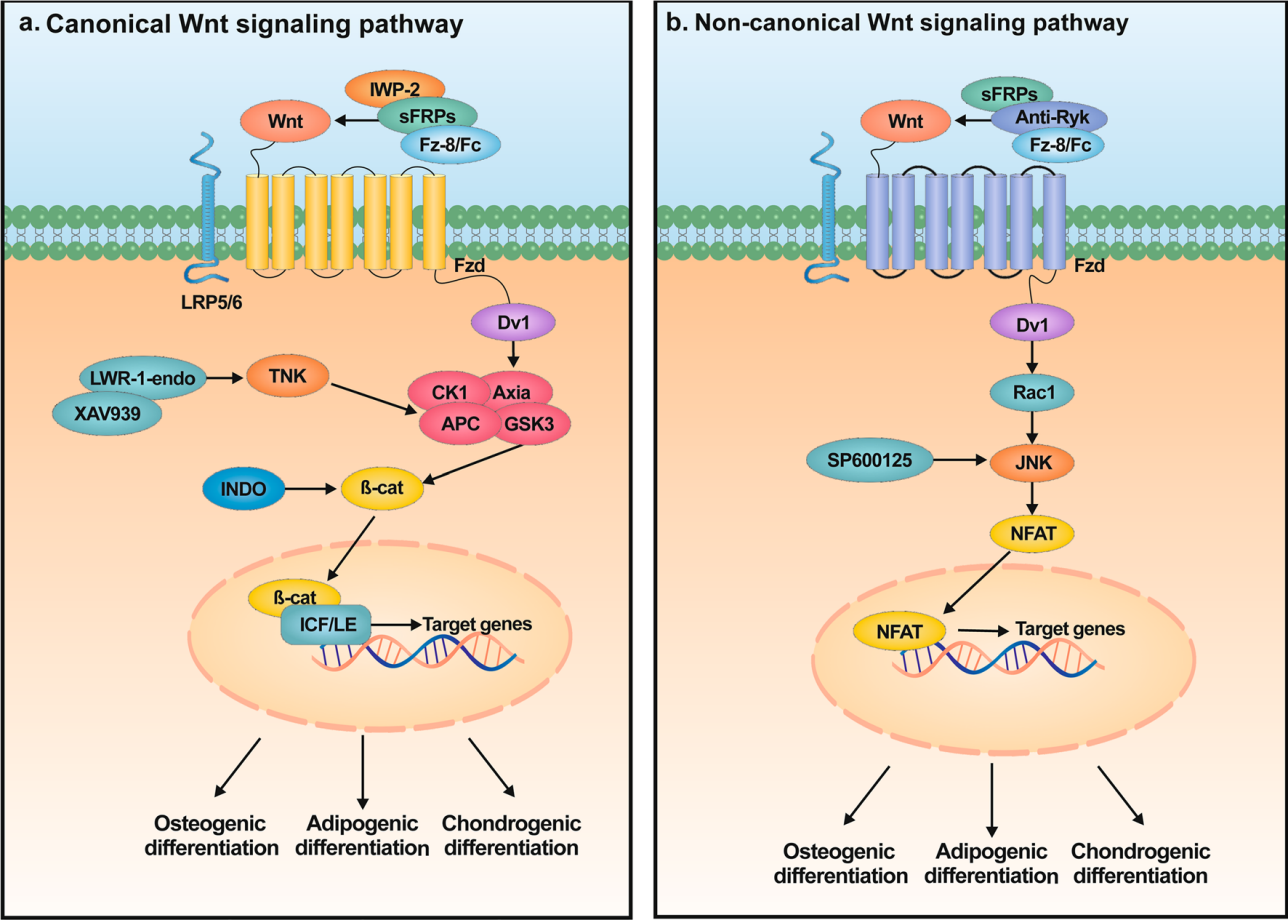
The biogenesis and function of Wnt signaling pathway. **a** Canonical Wnt signaling pathway. In the absence of Wnt signaling, β-catenin cannot accumulate in the cytoplasm as it is degraded by a multimeric protein complex consisting of APC, Axia, CK1 and GSK3). Receptor occupancy inhibits the kinase activity of the “destruction complex”, involving the actions of the Axia-binding molecule Dvl. Consequently, due to stabilization of β-catenin by disrupted Axia-mediated phosphorylation, β-catenin accumulates and travels to the nucleus where it engages the ICF/LE complex to mediate transactivation of target genes. **b** The non-canonical Wnt signaling pathway is initiated by Wnt binding to Fzd, then Dvl is recruited, followed by Rac1, JNK and NFAT activation and cytoskeletal rearrangement, and then NFAT is translocated to the nucleus to activate the expression of Wnt target genes

In summary, osteogenic differentiation, adipogenic differentiation and chondrogenic differentiation are in balance with each other to maintain the homeostasis of the organism. The disruption of the expression of specific miRNAs may lead to the development of bone-related diseases because of their dual roles. The depth of this discovery will also provide promising solutions for the clinical treatment of osteoarthritis, osteoporosis, and other diseases (Fig. 3).

## Modulation of signaling pathways in tri-lineage differentiation of C3H10T1/2

**Fig. 4:**
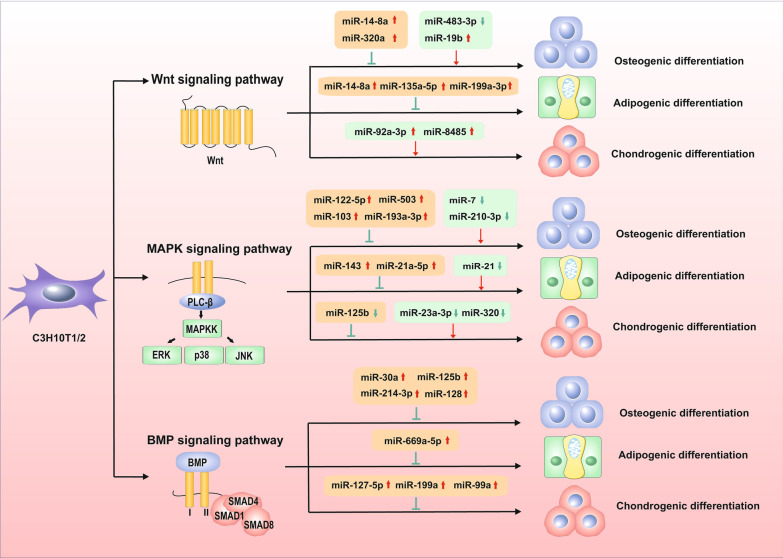
Wnt signaling pathway regulations in tri-lineage differentiation. The Wnt signaling pathway regulate the tri-lineage differentiation of C3H10T1/2 through canonical or non-canonical signaling pathway. Specific miRNAs have been confirmed to regulate the activation or inhibition of Wnt signaling pathway, which further acts on the downstream signal molecules, and then guide the commitments of osteogenic, adipogenic and chondrogenic differentiation of C3H10T1/2. “ → ”represents the upstream signal promoting the downstream signal, and “⊥”represents the downstream signal inhibiting the downstream signal. The red arrows refer to the promoting effects, while the green arrows and lines refer to suppressing effects

With the continuous progress of science and technology, the quality of people’s lives gradually improved, the phenomenon of aging of the population is becoming increasingly obvious, and the incidence of common diseases of the elderly is also showing a trend of increasing year by year, especially metabolic bone disease, osteoarthritis [17]. For another, it was found that BMP signaling pathway, Wnt signaling pathway, RANKL signaling pathway, NOTCH signaling pathway, NFAT signaling pathway and MAPK signaling pathway were involved in the regulation of bone metabolism [14, 15]. Among them, BMP signaling pathway, Wnt signaling pathway and MAPK signaling pathway have also been confirmed to be involved in inducing chondrogenic and adipogenic differentiation in C3H10T1/2, suggesting potential development space for targeted drug design in osteoarthritis, osteorheumatism and other diseases. Numerous miRNAs promote the differentiation of C3H10T1/2 by regulating the activation or inhibition of signaling pathways, and differences in their distribution and duration of action may lead to differences in results. Therefore, in the following passage, we mainly focus on the process of miRNAs regulating C3H10T1/2 differentiation through BMP pathway, Wnt pathway and MAPK pathway (Fig. 4).

### MAPK signaling pathway

#### Biogenesis of MAPK signaling pathway

MAPKs is an important signal transduction system that mediates cell reactions widely in eukaryotic cells. It is characterized by tyrosine phosphorylation after stimulated by cytokinin and growth factor polypeptides [90]. MAPK signaling pathway includes three core kinases, MAP3K, MAPKK and MAPK, in addition to upstream component MAP4K and downstream component MAPKAPK [91]. Activation of MAPK signaling pathway is through the execution of a conserved tertiary enzymatic cascade: Phosphorylated MAP3K activates MAPKK phosphorylation and finally activates MAPK phosphorylation [92]. Massive studies have found that MAPK signaling pathway plays a crucial regulatory role in the triadic differentiation of cells. Different miRNAs stimulate ERK signaling pathway and have different effects on osteogenic differentiation of cells. It has been found that both miR-9 and miR-10 play an important role in the differentiation of BMSCs into osteoblasts. Appropriate miR-9 can promote the differentiation of osteoblasts, while overexpression of miR-9 can inhibit the differentiation of osteoblasts, and miR-10 can inhibit the differentiation of osteoblasts [93]. In addition, other studies have shown that miR-205 can promote the differentiation of BMSCs into osteoblasts, while miR-217 has the opposite effect [94].

#### MAPK signaling pathway in tri-lineage differentiation

MAPK signaling pathways mainly include ERK1/2 pathway, JNK pathway, P38 pathway and ERK5 pathway, which is involved in the multidirectional differentiation of osteoblasts, adipocytes and chondrocytes. Substantial researches have been completed to explore multidirectional differentiation using C3H10T1/2 as experimental subjects (Table 5).

**Table 4:** miRNAs play a dual role in tri-lineage differentiation of C3H10T1/2

miRNAs	Osteogenic differentiation	Adipogenic differentiation	Chondrogenic differentiation	References
miR-223	↓	↑	–	[7]
miR-582-5p	↓	↑	–	[49]
miR-194	↑	↓	–	[52]
miR-30e	↓	↑	–	[8]
miR-199a	↑	–	↑	[86]
miR-210-3p	–	↓	↑	[87]
miR-21	↑	↑	–	[88]
miR-145-5p	–	↑	↓	[89]

“↑” refers to activation, “↓” refers to inhibition, and “–” refers to unrelated

MiRNA usually acts as an upstream signal molecule to regulate the expression of MAPK signal pathway, thus regulating the osteogenic differentiation of C3H10T1/2 cells. The mechanism is composed of multiple factors, and some miRNAs affect various signaling pathways of MAPK by activating or inhibiting intracellular phosphorylation activities. For instance, miR-193a blocks the phosphorylation of MAPK family proteins (p-JNK, p-ERK, p-p38), thus inhibiting osteogenic differentiation of C3H10T1/2 [95]. Some miRNAs directly interact with MAPK family proteins to regulate the induction of osteogenic. Huang et al. [96] found that miR-503 inhibited RANK expression by directly targeting RANK during osteoclast differentiation, while there was no significant change in the expression of p-Akt, p-p38, and p-ERK in Western blotting. Also, some non-coding RNAs also regulate osteogenic differentiation by competing with miRNAs for binding targets or directly binding inhibition. Zhu et al. [97] confirmed that circ_0001843 directly bound with miR-214, and the overexpression of miR-214 inhibited the phosphorylation of p38 and JNK, suppressing bone formation.

MAPK signaling pathway also plays a key role in regulating adipogenic differentiation in C3H10T1/2 cells. The differentiation of C3H0T1/2 cells into adipocytes is a complex physiological process that includes clonal expansion, growth arrest and terminal differentiation. MiRNAs indirectly participate in these adipogenic differentiation process by directly regulating the expression of MAPK family proteins. The same miRNA that acts at different times may have the opposite effect. For instance, when miR-143 is overexpressed during the clonal expansion stage, the adipogenic differentiation is inhibited, whereas the overexpression of miR-143 during the growth arrest stage or terminal differentiation stage promotes the adipogenic differentiation of C3H10T1/2 [98].

MiRNAs can also indirectly regulate cell chondrogenic differentiation through the MAPK signaling pathway. It is noteworthy that unlike osteogenic and adipogenic differentiation, miRNAs often co-regulate cytokines on the MAPK family proteins in chondrogenic differentiation. Surprisingly, interleukin is the most common among the cytokines involved in regulation. For instance, Sun et al. [99] discovered that miR-320c overexpression and CDK6 inhibition repressed IL-1β-induced expression of inflammatory factors and regulated the NF-kB signaling pathway. Meng et al. [100] found that IL-1β led to a significant reduction in miR-320 expression, while the overexpression of miR-320 down-regulates MMP-13 to inhibit adipogenic differentiation.

### Wnt signaling pathway

#### Biogenesis of Wnt signaling pathway

Wnt signaling is transmitted by a seven-channel family of transmembrane g protein-coupled acceptors (frizzled (Fzd)) and a family of arrow/Lrp co-receptors (e.g., Lrp5 and Lrp6) or Ryk or Ror transmembrane tyrosine kinases [109]. The binding of a given Wnt to Fzd receptors and co-receptors stimulates a variety of different endocellular signal cascades, separated into typical β-catenin-dependent pathways and atypical β-catenin-independent pathways [110, 111]. β-catenin is a vital transcription costimulator regulating genetic transcription in reaction to the Wnt signal pathway [112]. Generally, the cytoplasmic contents of β-catenin are maintained at low levels in cells not exposed to ligands through interaction with the β-catenin destruction complexes [113]. The binding of Wnt to the Fzd acceptor complexes leads to the phosphonation of the Lrp co-receptor and the recruiting and binding of GSK3β and Axin to ligand-acceptor complexes. These complexes are afterward endocytosed and suppressed by segregation into multi-vesicular nuclear endosomes, leading to the stabilization and cumulation of cytoplasm β-catenin [114]. Stabilized β-catenin moves into nuclei and interacts with lymphocyte enhancer factor/t-cell factor (Lef/Tcf) family high mobility group (HMG)-type transcriptional factors to activate the expressing of targeted genes, such as Lef1, Tcf7, Nkd2 and Axin2 [115–118]. The pathway of Wnt ligand activation is decided by various factors, like specific ligand-acceptor interplay, diverse acceptor/co-acceptor pairs or the existence of endocellular protein regulating β-catenin stimulation. Wnt signal transmission is remarkably regulated at several levels to guarantee the appropriate activities in normal developmental process and tissular homeostasis (Fig. 3).

#### Wnt signaling pathway in tri-lineage differentiation

The Wnt signal pathway is a conserved signaling axis involved in a variety of physiologic process like proliferative, differentiative, apoptotic, migratory, invasive and tissular homeostasis [119–121]. Recent studies on the tri-lineage differentiation of the Wnt signaling pathway have attracted extensive attention [122]. Osteoblasts generate bone matrix protein with an overall life span of 2–3 months [123]. Finally, they apoptose and exist on bone surfaces as endosteal cells embedded in autocrine bone matrix proteins or differentiate into osteoblasts. Osteoblasts are stemmed from non-differentiated mesenchymal cells and can also differentiate into chondrocytes, adipose cells, myoblasts and fibroblasts. The differentiation of progenitor cells into tissue-specific cells is modulated by tissue-specific transcriptional factors. Substantial researches have been completed to explore multidirectional differentiation using C3H10T1/2 as experimental subjects (Table 6), but there remains room to investigate the mechanisms of the Wnt signal pathway.

**Table 5:** Modulation of MAPK signal pathway in tri-lineage differentiation of C3H10T1/2

Differentiation	Target	Expression	Expression of MAPK	Distribution	Function	Experiment	Sample	References
Osteogenic differentiation	miR-122-5p	↑	↓	Exosomes	Overexpressed miR-122-5p inhibits the RTK/Ras/MAPK signaling pathway, thereby down-regulating SPRY2 and promoting osteogenesis	In vivo and in vitro	Mice/80	[101]
	miR-7	↓	↑	Cytoplasm	The down-expression of miR-7 up-regulated GDF5, promoting the phosphorylation of Smad1/5/8 and p38 MAPK to facilitate osteogenic differentiation	In vivo	–	[102]
	miR-503	↑	↓	Cytoplasm	miR-503 down-regulate RANK to suppress the MAPK and AKT pathways, which resulted in decreased expression of osteoclastogenesis-related markers	In vivo	–	[96]
	miR-103	↑	↓	Cytoplasm	miR-103 inhibit the activation of mechanical kinase ROCK1 and p-Erk1/2 in the MAPK signaling pathway, thereby inhibiting the classical osteogenesis-related Wnt/β-catenin pathway	In vivo and in vitro	Mice/40	[103]
	miR-210-3p	↓	↑	Nucleus	miR-210-3p suppresses osteogenic differentiation through targeting KRAS and suppressing the MAPK signaling; KRAS overexpression could reverse the suppressive effects of miR-210-3p overexpression	In vivo	–	[104]
	miR-193a-3p	↑	↓	Cytoplasm	miR‐193a‐3p binds to Map3k3 mRNA, which results in down-regulation of Map3k3 expression, suppressing bone formation	In vivo and in vitro	Mice/60	[105]
Adipogenic differentiation	miR-143	↑	↓	Cytoplasm	The overexpression of miR-143 blocks the MAP2K5 ERK5 signaling pathway, so the ERK5-mediated phosphorylation of PPAR-γ is reduced	In vivo	–	[98]
	miR-21a-5p	↑	↓	Nucleus	miR-21a-5p inhibited BPA induced adipocyte differentiation by targeting map2k3 through MKK3/p38/MAPK	In vivo	–	[106]
	miR-21	↓	↑	Cytoplasm	miR-21 modulated ERK-MAPK signaling activity by repressing SPRY2 expression, a known regulator of the receptor tyrosine kinase (RTK) signaling pathway, to affect the duration and magnitude of ERK-MAPK activity	In vivo	–	[88]
Chondrogenic differentiation	miR-23a-3p	↓	↑	Cytoplasm	miR-23a-3p regulated by lncRNA SNHG5 suppresses the chondrogenic differentiation via targeting activating the JNK/MAPK/ERK pathway	In vivo	–	[107]
	miR-320	↓	↑	Cytoplasm	miR-320 promoted the activity of a reporter construct containing the 3'-UTR and inhibited MMP-13 expression	In vivo	–	[100]
	miR-125b	↓	↓	Nucleus	The down-regulation of miR-125b induced apoptosis and suppress other differentiation via BMPR2	In vivo and in vitro	Mice/40	[108]

It has been shown that the Wnt/β-catenin signal pathway participates in the osteogenic differentiation of C3H10T1/2 cells. The stimulation of the Wnt/β-catenin signal pathway facilitates osteogenetic differentiation and the inhibition of this pathway induces osteoclast differentiation [124]. For instance, Zhang et al. [125] claimed that the overexpression of miR-335-5p up-regulated the expressions of Runx2 and Osx proteins to induce osteogenic differentiation in mice, and also, miR-335-5p down-regulated the expression of DKK1 protein to support bone formation. Kureel et al. [126] discovered that the overexpression of miR-376c promotes β-catenin transactivation by targeting Wnt3 and ARF-GEF-1, and thus inhibiting osteogenic differentiation. By participating in the activation of osteogenic C3H10T1/2 differentiation promoted by the Wnt/β-catenin signal path, miRNAs inhibit the differentiation of C3H10T1/2 to chondrogenic and adipogenic cell lines, prevent the apoptosis of mature osteoblasts and suppress osteoclast differentiation, thereby preventing osteoporosis.

Recent researches have also revealed that the Wnt signal pathway exerts a key effect on the adipogenic differentiation of C3H10T1/2 cells. Research shows that the overexpression of miR-210-3p promoted the osteogenic differentiation and inhibited the adipogenic differentiation of C3H10T1/2, which may be related to Wnt signaling pathway [127].

At present, massive studies have focused on the role of Wnt/β-catenin signal pathway in chondrogenic differentiation of stem cell, but there are still few studies on C3H10T1/2 cells as experimental targets. The clarification of this mechanism is conducive to the innovation of clinical treatment methods for osteoarthritis and cartilage damage diseases, and the mechanism of Wnt signal pathway in chondrogenesis has more space for exploration.

### BMP signaling pathway

#### Biogenesis of BMP signaling pathway

Bone morphogenetic protein (BMP) is an important member of TGF-β superfamily [136]. BMPs participate in biological functions in the form of active dimer, and the 7 cysteine residues in the carboxyl terminal of its precursor show a high degree of conservation [137]. They are produced and secreted to the functional site by the polymerization of disulfide bonds after proteolysis guided by the cysteine residues mentioned above. At the same time, type II receptor (BMPR2) activates type I receptor (BMPR1) and further transmits signal to Smad molecules in the cell [138]. Smad proteins play a key role in the transfer of BMP and TGF-β signals from the cell membrane to the nucleus. A large number of studies have shown that activation of BMP signaling channels promotes osteoblast formation. BMP-Smad signaling up-regulates Runx2 expression, which interacts with Smad1/5/8 to activate transcriptional downstream genes, thereby promoting osteogenic differentiation. Matsubara et al. [139] found that Runx2 and Mxs2 play an important role in the activation of Osterix by the classic BMP signaling pathway, while TGF-β-induced Smad3 blocks Runx2. Sheu et al. found that GIT1 knockout mice had delayed fracture healing. Further studies found that GIT1 knockout mice had decreased expression of BMP-2 in MSC, Smad1/5/8 phosphorylation, Runx2 expression and bone formation ability, resulting in delayed fracture healing BMP-3 inhibits BMP-2 and BMP-4, thereby inhibiting the BMP-Smad signaling pathway, thereby inhibiting osteogenic differentiation SMURF1 blocks early osteogenic differentiation by acting on Runx2 [140]. In addition, inhibitory Smads such as Smad6 and Smad7 play a down-regulatory role on osteoblast differentiation. A study constructed adenoviral vectors expressing Smad6 or Smad7, infected them into C2C12 cells and stained the cells with anti-Smad5 antibodies. It was found that Smad6 or Smad7 could prevent the nuclear translocation of Smad5 induced by BMP-6, suggesting that BMP signaling pathways in mammals could be inhibited from inhibitory Smads by blocking the nuclear translocation of receptor-regulated Smads [139].

#### BMP signaling pathway in tri-lineage differentiation

Numerous studies demonstrate that BMP-2, 4, 5, 7 can effectively promote the tri-lineage differentiation of C3H10T1/2 (Table 7). There is BMPR on the osteoblastic membrane, which is activated by binding to BMP produced by mechanical stimulation and then act on downstream Smad proteins in the cytoplasm.

**Table 6:** Modulation of Wnt signal pathway in tri-lineage differentiation of C3H10T1/2

Differentiation	Target	Expression	Expression of Wnt	Distribution	Function	Experiment	Sample	References
Osteogenic differentiation	miR-148a	↑	↓	Cytoplasm	miR-148a suppressed osteogenic differentiation via targeting the Wnt5a/Ror2 pathway	In vivo	–	[128]
	miR-483-3p	↓	↑	Cytoplasm	miR-486-3p targets CTNNBIP1, thus activating the Wnt/β-catenin signaling pathway to promote osteogenesis of C3H10T1/2	In vivo and in vitro	Mice/42	[129]
	miR-320a	↑	↓	Nucleus	miR-320a inhibited the activity of Wnt/β-catenin signaling pathway through CTNNB1 inhibition, which cause the inhibition osteogenic differentiation in osteoporosis	In vivo and in vitro	Mice/48	[130]
	miR-19b	↑	↑	Exosome	miR-19b represses the expression of WWP1 or Smurf2 and elevates KLF5 expression through the Wnt/β-catenin signaling pathway, thereby facilitating osteogenic differentiation	In vivo	–	[131]
Adipogenic differentiation	miR-148a	↑	↓	Cytoplasm	miR-148a promoted adipogenic differentiation via targeting the Wnt5a/Ror2 pathway	In vivo	–	[128]
	miR-199a-3p	↑	↓	Cytoplasm	The overexpressed miR-199a-3p regulates adipogenesis by suppressing the activity of KDM6A/Wnt signaling	In vivo and in vitro	Mice/20	[132]
	miR-135a-5p	↑	↓	Cytoplasm	miR-135a-5p suppresses 3T3-L1 preadipocyte differentiation and adipogenesis through the activation of canonical Wnt/β-catenin signaling	In vivo	–	[133]
Chondrogenic differentiation	miR-92a-3p	↑	↑	Nucleus	Overexpressed miR-92A-3p targets Wnt5a to enhance chondrogenic differentiation and inhibits cartilage degradation	In vivo	–	[134]
	miR-8485	↑	↑	Exosome	miR-8485 targeted GSK3B to repress GSK3β expression and targeted DACT1 to induce p-GSK3β, activating Wnt/β-catenin pathways	In vivo	–	[135]

**Table 7:** Modulation of BMP signal pathway in tri-lineage differentiation of C3H10T1/2

Differentiation	Target	Expression	Expression of BMP	Distribution	Function	Experiment	Sample	References
Osteogenic differentiation	miR-30a	↑	↓	Cytoplasm	The overexpression of miR-30a led to expression of an early osteogenic marker and a reduction in Runx2 expression, thus inhibiting the osteogenic differentiation of C3H10T1/2	In vitro and in vivo	Mice/24	[45]
	miR-125b	↑	↓	Cytoplasm	The over and interferential expression of miR-125b down-regulate for Cbfβ protein in C3H10T1/2 cells and the overexpression decrease the mRNA levels of three osteoblastic marker genes, ALP, OCN, OPN by BMP-2-induced	In vitro and in vivo	Mice/40	[53]
	miR-214-3p	↑	↓	Nucleus	miR-214-3p could prevent C3H10T1/2 osteogenic differentiation by targeting BMP-2 and blocking BMP-TGFβ axis	In vitro	–	[146]
	miR-128	↑	↓	Cytoplasm	The overexpression of miR-128 promoted osteogenic differentiation of C3H10T1/2 by up-regulating ALP, matrix mineralization, mRNA, and protein levels of BMP-2	In vitro	–	[147]
Adipogenic differentiation	miR-669a-5p	↑	↓	Cytoplasm	The overexpression of miR-669a-5p promotes adipogenic differentiation and causes browning of C3H10T1/2 cells	In vitro	–	[148]
Chondrogenic differentiation	miR-127-5p	↑	↓	Cytoplasm	LncRNA DNM3OS regulates GREM2 via miR-127-5p to suppress early chondrogenic differentiation of C3H10T1/2 under hypoxic conditions	In vitro	–	[149]
	miR-199a	↑	↓	Cytoplasm	miR-199a inhibits Smad1/Smad4-mediated transactivation of target genes, and that overexpression of Smad1 completely corrects miR-199a-mediated repression of early chondrogenesis	In vitro and in vivo	Mice/48	[142]
	miR-99a	↑	↓	Cytoplasm	miR-99a knockdown promoted proteoglycan deposition and increased the expression of ACAN and COL2A1 during early chondrogenic differentiation	In vitro	–	[150]

Smad1 and Smad5 are closely related to osteogenesis differentiation. The serine residues at the end of Smad1, 5 and 8 are phosphorylated by BMPR receptor, and then, two or one R-Smad and one Smad4 enter the nucleus as heterotrimers or heterodimers, which act on and up-regulate the expression of osteoblast specific transcription factors such as Runx2 and Osterix [139]. For instance, Qin et al. [141] demonstrate that miR-494 directly targeted BMPR2 and Runx2, promoting the process of BMP-2-induced osteogenetic differentiation. Furthermore, BMP signaling pathway plays an important role in chondrogenic differentiation of C3H10T1/2. As an upstream signal channel, miRNAs often inhibit the expression of BMP signaling pathway and thus inhibit chondrogenic differentiation. Lin et al. [142] discovered that miR-199a inhibits Smad1/Smad4-mediated transactivation of target genes, while the overexpression of Smad1 inhibits the expression of miR-199a at early chondrogenesis.

Notably, BMP and classical Wnt pathways can not only regulate osteogenesis, but also regulate each other at different stages from pathway activation to biological effects. Rawadi et al. [143] found that after DKK1 was used to block classical Wnt signaling pathway, BMP signaling pathway-induced ALP activity was inhibited, and BMP signaling pathway-induced extracellular matrix mineralization was weakened, suggesting that BMP signaling pathway’s effect on extracellular matrix mineralization was partially mediated by autocrine or paracrine circuitry of classical Wnt signaling, and BMP signaling pathway activation could enhance Wnt3 and Wnt3a. Zhang et al. [144] also proved that the activation of BMP signaling pathway induces the expression of Wnt ligand protein and receptor, and enhances the activity of classical Wnt signaling pathway. Yang et al. [145] found that exogenous BMP can inhibit the expression of β-Trep and increase the non-phosphorylated β-catenin in the classical Wnt signaling pathway by activating BMP signaling pathway. It can be concluded that BMP signaling pathway regulates the interaction between BMP and Wnt signaling pathway in the process of osteogenic differentiation by reducing the degradation of β-catenin and increasing the ligand protein of classical Wnt signaling pathway. The interaction between BMP and classical Wnt signaling pathway in different stages can achieve the balance of osteogenic differentiation.

### The others

In addition to MAPK signaling pathway, Wnt signaling pathway and BMP signaling pathway, many related signaling pathways also play crucial roles in the differentiation of C3H10T1/2.

RANKL signaling pathway, NOTCH signaling pathway and Hedgehog signaling pathway are recognized as one of the signaling channels involved in bone metabolism, which has been demonstrated to promote or inhibit the differentiation of bone marrow mesenchymal stem cells, bone marrow-derived macrophages, human embryonic stem cells and other cells [151–153]. We were surprised to find that these signaling pathways also play a similar regulatory role in the osteogenic differentiation of C3H10T1/2. Bae et al. [154] claimed that miR-34c targets multiple components of the Notch signaling pathway, including Notch1, Notch2 and Jag1 in a direct manner, and influences osteoclast differentiation in a non-cell-autonomous fashion. Zhong et al. [155] discovered that the overexpression of miR-196a repressed GNAS expression by activating the Hedgehog signaling pathway, thus promoting the osteogenic differentiation of C3H10T1/2. Research have also found that miR-506-3p has a high affinity for NFATc1, and the up-regulation of miR-506-3p in mice can selectively inhibit NFATc1, thereby inhibiting the secretion of various downstream bone resorptive enzymes, and thus inhibiting the osteogenic differentiation of C3H10T1/2 [156].

Some signal pathways are highly correlated with the adipogenic differentiation of C3H10T1/2. Tang et al. [157] found that miR-206-3p reduced the phosphorylation of c-Met downstream effector Akt in the PI3K/Akt signaling pathway, and the inhibition of c-Met expression suppressed the adipogenic differentiation of C3H10T1/2, and the recovery of c-Met expression could counteract the inhibitory effect of miR-206-3p. Mi et al. [158] discovered that miR-139-5p blocked adipogenesis of C3H10T1/2 via directly targeted the 3' untranslated regions of NOTHCH1 and IRS1 mRNAs, while the up-regulation of NOTHCH1 and IRS1 partially restored the inhibitory effect of miR-139-5p.

Many signaling pathways are involved in chondrogenic differentiation of stem cells, such as NF-κB signaling pathway, PI3K/Akt signaling pathway and JAK/STAT signaling pathway [159–161]. However, studies based on C3H10T1/2 are relatively scarce, suggesting that this is still a blank field to be developed. Zhang et al. [76] found that the overexpression of miR-199b-5p enhanced the expression of chondrocyte-specific markers of SOX9, Aggrecan and Col2a1, thereby promoting chondrogenic differentiation of C3H10T1/2. Interestingly, up-regulation of miR-199b-5p also inhibited cell proliferation, while inhibition of miR-199b-5p significantly promoted the proliferation of C3H10T1/2, but decreased its chondrogenic differentiation.

In conclusion, signaling pathways (PI3K/Akt signaling pathway, NOTCH signaling pathway, Hedgehog signaling pathway and other key signaling pathways) can directly or indirectly act on key transcription factors such as Runx2 or Osterix and interact with each other, thus forming an intricate regulatory network that cooperatively participates in the tri-lineage differentiation of C3H10T1/2. We believe that with the further development of relevant research, the complete molecular mechanism of osteogenic, adipogenic and chondrogenic differentiation of stem cells is expected to be thoroughly uncovered, so as to provide a new theoretical basis and therapeutic targets for the effective clinical prevention and treatment of osteoporosis, ectopic ossification and other diseases.

## Conclusion

In recent years, with the in-depth discoveries and researches of C3H10T1/2, its extensive sources, outstanding proliferation ability, multi-differentiation potential and satisfactory biocompatibility with various 3D scaffold materials display its exciting potential in the treatment of bone defects. Further researches have found that miRNAs are pivotal for C3H10T1/2 cell tri-lineage differentiation.

miRNAs exert an important effect on the balance among osteoblast, adipogenic and chondrogenic differentiation, which provides a promising treatment regimen for osteoporosis, osteoarthritis and other diseases. In addition, miRNAs can also extensively participate in the regulation of Wnt signaling pathway, MAPK signaling pathway and BMP signaling pathway by acting directly on specific proteins and indirectly competing against a target gene, thus affecting the tri-lineage differentiation of C3H10T1/2. It is worth noting that when the same miRNAs act on the above three signaling pathways, they may have dual or anti-temporal effects. For instance, the Wnt signaling pathway has been evidenced to facilitate the osteogenetic differentiation of C3H10T1/2 and play a dual role in its adipogenic differentiation, whereas chondrogenic differentiation may be inhibited by such pathway. The same miRNA affecting the expression of MAPK signal pathway at the clonal expansion stage inhibited the adipogenic differentiation of C3H10T1/2, while it plays a completely opposite role in the growth arrest stage or terminal differentiation stage. In addition, BMP signaling pathway and Wnt signaling pathway often promote and influence each other during osteogenic differentiation of C3H10T1/2.

Massive studies have deeply revealed the mechanisms of miRNAs with regard to the regulation of C3H10T1/2 differentiation and the balance among osteoblast, adipogenic and chondrogenic differentiation. However, the understanding of the functional miRNA biogenesis and localization, and especially the understanding pertaining to how tissue-specific expression facilitates their specific modulatory patterns in different biological processes are elusive. Moreover, though there is a certain basis for the research on the mechanism of miRNA-induced cell differentiation, few people apply this theoretical knowledge to develop miRNA-targeted molecular drugs for bone diseases. Safety and efficacy are the main challenges in the design and development of miRNA-targeted drugs and are also the main reasons for the failure of early drug development. In addition, drug resistance is more likely to occur in targeted drug therapy compared with conventional treatment regiments, and the therapeutic effect in the middle and later stages is still unclear. However, it is of great interest to apply the discoveries obtained from miRNA regulation to clinical treatments. By optimizing the route of administration and time of action, miRNA-targeted drugs may also achieve better therapeutic effects. We sincerely hope that further researches on the regulatory mechanisms of miRNAs may lay the ground for the clinical therapies of bone defect-related illnesses.

## Data Availability

Not applicable.

## Abbreviations

miRNAs: MicroRNAs
3′ UTR: 3′ untranslated region
Ago2: Argonaute
RBP: RNA-binding protein
pri-miRNA: Primary miRNA transcripts
RISC: RNA-induced silencing complex
ECM: Extracellular matrix
WAT: White adipose tissue
BAT: Brown adipose tissue
PPARγ: Peroxisome proliferator-activated factor receptor γ
PGD-1α: PPARγ coactivator 1α
C/EBPα: CCAAT/enhancer-binding protein A
AP2: Adipocyte protein 2
TGF-β3: Transforming growth factor-β3
OP: Osteoporosis
Lef/Tcf: Lymphocyte enhancer factor/t-cell factor
HMG: High mobility group
Runx2: Runt-associated transcriptional factor 2
ALP: Alkaline phosphatase
